# Differentiation of fertility attitudes and its influencing factors among married reproductive-aged women: an empirical analysis in Shandong Province, China

**DOI:** 10.3389/fpubh.2026.1840600

**Published:** 2026-06-10

**Authors:** Zihan Zhou, Yanli Li, Mengjun Cao, Hongyan Hao, Ying Zhao, Na Liu, Qiang Wang

**Affiliations:** 1 College of Public Health, Shandong Second Medical University, Weifang, Shandong, China; 2 Library, Shandong Second Medical University, Weifang, Shandong, China; 3 Department of Performance Management, Qingdao Central Hospital, University of Health and Rehabilitation Sciences (Qingdao Central Hospital), Qingdao, Shandong, China; 4 Department of Epidemiology, Shandong Second Medical University, Weifang, Shandong, China

**Keywords:** fertility attitudes, fertility policy, influencing factors, married women, reproductive-aged women, universal two-child policy

## Abstract

**Objectives:**

Under the universal two-child policy, this study investigates the differentiation of fertility attitudes among married reproductive-aged women in Shandong Province, and analyzes the key factors associated with traditional and emerging fertility attitudes.

**Methods:**

A cross-sectional study was conducted among married reproductive-aged women aged 18–45 in Shandong Province. Univariate analysis and binary logistic regression were used to analyze the differentiation of fertility attitudes and their main correlated factors.

**Results:**

A total of 1,782 valid questionnaires were collected. Logistic regression analysis showed that traditional fertility attitudes were positively associated with factors such as low educational attainment, having family health risks, family economic status, high fertility expectations from husbands, parents, parents-in-laws, society, and community support (*p* < 0.05). In contrast, emerging fertility attitudes showed a significant negative association with high older adult care and childcare expenditures, strong parental and societal expectations, and perceived inadequacy of policy reimbursement amounts and community support (*p* < 0.05).

**Conclusion:**

Fertility attitudes among married reproductive-aged women in Shandong Province present a complex interplay with the coexistence of both traditional and emerging perspectives. Traditional fertility attitudes are primarily related to health risks, intergenerational and social expectations, whereas emerging fertility attitudes are associated with familial economic pressure, external societal expectations, and perceived inadequacy of policy support.

## 1 Introduction

Globally, many countries and regions are experiencing a persistent decline in fertility rates. Data from the United Nations Department of Economic and Social Affairs (UN DESA) indicate that the global total fertility rate (TFR) fell from approximately 3.2 in 1990 to 2.3 in 2021 and is projected to continue falling (1). China is no exception to this trend. Research shows that China’s TFR has significantly decreased from 2.2 in 1990 to 1.3 in 2020 (2). To address the demographic challenge of population aging and low fertility, the Chinese government implemented the universal two-child policy in 2016. This indicates that China’s fertility policy has shifted to a new stage of comprehensively lifting restrictions and encouraging childbirth (3). However, although the policy had a short-term significant effect in the initial stage of implementation, it failed to achieve the long-term increase in fertility rates (4–6). Previous studies indicate that fertility intention is influenced by a variety of factors (7). Aside from fertility policy, fertility intention remains constricted by multiple factors such as economy, society, and culture (8–10). In this context, understanding the fertility intentions and attitudes among the reproductive-aged population, particularly among women who bear the primary childbearing responsibility, is of great significance for predicting fertility trends, evaluating policy effectiveness, and building effective support systems.

Fertility attitudes are important psychological and cultural factors influencing an individual’s willingness and behavior to have children. They are not static but constantly evolve in the course of social changes (11). Existing studies generally observe that the fertility attitudes of reproductive-aged Chinese women are transitioning from traditional to modern perspectives. For instance, there is a shift from emphasizing “continuing the family line”, “more sons, more happiness”, and “raising sons for old age security” to focusing on the quality of children and personal development (12, 13). However, this transition is not a straightforward replacement. In many situations, traditional and emerging fertility attitudes may coexist, interweave, or even conflict, creating a state of complex conceptual differentiation (14). Most existing studies focus on investigating fertility intentions and their influencing factors, or the dominance of a single conceptual type. Yet, there is still a lack of in-depth empirical analysis on the differentiation of fertility attitudes and the specific influencing factors that drive or inhibit the formation of traditional and emerging fertility attitudes.

In China, the family plays a pivotal role in shaping individual fertility decisions. Studies show that fertility expectations from spouses, parents, and parents-in-laws constitute a significant source of familial pressure, profoundly influencing individual women’s childbearing choices (15, 16). Concurrently, the rapidly rising childcare costs, the persistently high housing and education expenses, and the dual burden of supporting both aging parents and young children have become economic barriers that suppress fertility intentions (17, 18). Furthermore, although the government has introduced supportive policies, including cash subsidies and childcare services, whether these policies can be perceived at the grassroots level and truly alleviate the fertility concerns of families remains a question to be verified (19).

As a populous province in China, Shandong has a certain typicality in its social and economic structure and cultural attitudes for demographic research (20, 21). Shandong is China’s second most populous province and has experienced fertility decline similar to the national trend, making it an important region for fertility research (22). As the home of Confucius and Mencius, Shandong is deeply influenced by traditional Confucian culture, which emphasizes lineage continuation, filial piety, and the value of having children (21). Since the 1960s, Shandong Province has played a leading role in the standardized implementation of the national family planning policy (22). Furthermore, as a major economic province, Shandong has experienced a large-scale expansion of higher education in recent years, which may make well-educated women more receptive to emerging concepts that differ from traditional marriage and fertility attitudes (22). These characteristics make Shandong a “typical” case for observing traditional fertility attitudes. Taking Shandong as a case study, this research investigates the differentiation of fertility attitudes and its influencing factors among married reproductive-aged women under the universal two-child policy context. And this study may provide empirical evidence for constructing a more targeted fertility support policy system, thereby contributing to the modernization of fertility attitudes and the promotion of long-term balanced population development.

## 2 Materials and methods

### 2.1 Definition of participants

This study adopted a cross-sectional design and was conducted in Shandong Province in 2024 through a questionnaire survey. The research subjects were married women of childbearing age aged 18 to 45 in Shandong Province. The age range of “young adults of reproductive age” as stipulated by Chinese standards is generally 18 to 49 years old (9). Therefore, a minimum age limit of 18 years old was set. Studies based on China’s long-term population data show that the cohort fertility rate (CFR) of women aged 45–49 has dropped to an extremely low level (1.62 in 2015) (23), indicating that the contribution of this age group to the overall fertility rate has become very limited. Therefore, the upper age limit for this study is set at 45 years old.

The inclusion criteria: (1) women of reproductive age (18–45 years old) with household registration in Shandong Province. (2) Married women. (3) Women without fertility disorders. (4) Women who voluntarily participated in the survey. (5) Women who can independently complete the survey questionnaire. The exclusion criteria: (1) women under the age of 18 or over 45. (2) Unmarried, divorced or widowed women. (3) Women who are not registered in Shandong Province. (4) Women who are unable or unwilling to complete the questionnaire independently.

### 2.2 Sample size

The sample size calculation formula for cross-sectional studies was adopted in this research (24).

$$n=\frac{Z_{a/2}^{2}*p*(1-p)}{d^{2}}$$

Here, *n* is the required sample size, Ζ_α/2_ is the standard normal value corresponding to the desired confidence level, *p* is the estimated proportion and d is the allowable margin of error. This study adopted a 95% confidence level (Ζ_α/2_ = 1.96). Referring to similar studies (25), the proportion of fertility intention was conservatively estimated as *p* = 0.5 in order to maximize the sample size requirement. The allowable margin of error (*d*) was set at 0.03. Substitute the above values into the formula:

$$n=\frac{1.96^{2}\times 0.5\times (1-0.5)}{0.03^{2}}\approx 1,067$$

Preliminary calculations indicated a minimum required sample size of 1,067. Through a multi-stage sampling and data collection, a total of 1,813 questionnaires were initially retrieved. After strict quality control, questionnaires that had incomplete information, contained logical contradictions, or failed to meet the inclusion criteria were eliminated. Then a total of 1,782 valid questionnaires were obtained, resulting in an effective recovery rate of 98.3%. This sample size exceeds the theoretical minimum sample size (1,067), fully satisfying the requirements for subsequent statistical analyses.

### 2.3 Sampling method

A multi-stage sampling approach was employed. Firstly, the 16 prefecture-level cities of Shandong Province were categorized into three distinct strata based on a comprehensive consideration of socioeconomic and cultural characteristics. Secondly, three cities were randomly selected from each stratum, yielding a total of nine sample cities. Thirdly, one county-level city or district was randomly chosen from each of the nine sample cities as the primary sampling unit, resulting in nine district- or county- level units. Fourthly, one sub-district and one township were further randomly selected as secondary sampling units from each selected district- or county-level unit, resulting in nine sub-districts and nine townships being retained. Fifthly, one urban community and one administrative village were randomly drawn from each sub-district and township unit as the final survey site, respectively, resulting in a total of 18 final survey sites (9 urban communities and 9 administrative villages). Finally, a cluster sampling was used at the designated survey sites. Eligible reproductive-aged women who met all inclusion criteria were invited to complete the survey independently during their visits to local healthcare centers or via community residential rosters. For the smallest sampling unit, a minimum of 90 individuals was required. If the number of eligible women in a final survey site did not meet this requirement, an additional randomly selected village or community within the same sampling unit was added.

Approximately 90–110 eligible participants were included in the final analytic sample from each final survey site, yielding 1,813 respondents in total, among whom 1,782 questionnaires were valid for analysis.

### 2.4 Questionnaire design

Data were collected using a self-designed structured questionnaire. The questionnaire primarily consisted of three parts.

The first part captured the participants’ basic demographic and socioeconomic information, including residence, age, educational attainment, marital status, occupation, health status, family structure, and so on.

The second part measured the key dependent variable in this study: fertility attitudes. Fertility attitude is a broad sociological concept. In this study, it refers to individuals’ evaluative orientations, values and cultural beliefs toward childbearing, which are conceptually distinct from fertility intentions (future fertility plans), decisions (fertility choices under constraints) or behaviors (realized fertility outcomes). Based on the sociological literature on Chinese fertility culture and attitudes (26–28), traditional fertility attitudes were defined as value orientations that emphasize lineage continuation, old-age security through offspring, and the positive value of having multiple children. Therefore, three questionnaire items were used to operationalize this construct: “In your perception, does the traditional fertility attitude of ‘raising sons for old-age security’ influence your choice to have a second child?”, “In your perception, does the traditional fertility attitude of ‘continuing the family line’ influence your choice to have a second child?”, “In your perception, does the traditional fertility attitude of ‘more sons, more happiness’ influence your choice to have a second child?”. Each item was rated on a five-point Likert scale (1 = no impact, 5 = very strong impact). A total score for traditional attitudes was calculated by summing the scores of these three items. Similarly, based on the literature in the context of contemporary China (26, 28), emerging fertility attitudes were defined as a preference for quality-oriented childrearing that prioritizes high investment in a small number of children over maximizing the number of children. Due to the exploratory nature of this study, emerging fertility attitudes were measured using a single item from the self-report questionnaire: In your perception, do emerging fertility attitudes such as ‘top-tier child-rearing’ or ‘elite-style child-rearing’ influence your choice NOT to have a second child? The score for emerging fertility attitudes was reverse-scored (i.e., 1 was converted to 5, and so forth). Subsequently, the total scores for both types of attitudes were dichotomized based on their respective medians. Scores above the median indicated holding that particular fertility attitude, while scores below the median indicated not holding it (29). That is, a total score ≥7.5 on the three traditional items indicated holding traditional fertility attitudes, and a score ≥2.5 on the single emerging item indicated holding emerging fertility attitudes.

The third part of questionnaire systematically included a range of potential independent variables affecting fertility attitudes. These comprised perceived evaluations of personal and family members’ health status; perceived impact levels of economic factors such as family financial status, older adult care expenses, and childcare costs for a second child; and socio-psychological factors like fertility expectations from husbands, parents-in-laws, parents, colleagues, friends, relatives, and society. These variables were also measured as categorical variables or using five-point scales. The overall questionnaire design aimed to comprehensively and systematically explore the complex factors influencing the formation of fertility attitudes among married reproductive-aged women.

The reliability and validity of the employed fertility attitude scales were tested. For the traditional fertility attitude scale (containing the three items mentioned above), the Cronbach’s *α* coefficient was 0.941, indicating excellent internal consistency. Exploratory factor analysis (EFA) showed a Kaiser-Meyer-Olkin (KMO) value of 0.764, and Bartlett’s test of sphericity was significant (*p* < 0.001), confirming the data’s suitability for factor analysis. As for the emerging fertility attitude measured by a single item, its validity was assessed through criterion-related validity. Analysis revealed that the score for emerging fertility attitudes (after reverse scoring) was significantly negatively correlated with the total score for traditional fertility attitudes (*p* < 0.001). This finding aligns with theoretical expectations, suggesting that this measurement effectively differentiates from traditional attitudes and demonstrates acceptable validity.

### 2.5 Statistical analysis

All collected data were managed and analyzed using Stata 18.0. The distribution of categorical variables, presented as percentages, described the proportion of married reproductive-aged women holding traditional versus emerging fertility attitudes across different subgroups. Associations between these categorical variables and fertility attitudes were examined using the chi-square (*χ^2^*) test. Binary logistic regression was performed to identify the key factors influencing the formation of fertility attitudes among married reproductive-aged women. A *p*-value of less than 0.05 was considered statistically significant.

### 2.6 Quality control

A series of quality control measures were implemented to ensure data quality and reliability. All investigators were uniformly trained and assessed to standardize procedures. Participants were selected via random sampling to reduce selection bias. Data accuracy was ensured through double-entry verification.

### 2.7 Ethics approval

The study was conducted in accordance with the ethical standards of the Declaration of Helsinki (1964, most recently revised in 2013) and was approved by the Ethics Review Committee of Shandong Second Medical University (No. 2023YX-139). All participants provided written informed consent prior to inclusion.

## 3 Results

### 3.1 Baseline characteristics

Supplementary Table 1 presents the baseline characteristics of 1782 participants. The majority were aged 30–39 years (54.0%), lived in urban areas (76.4%), held a master’s degree or above (74.7%), and were married with children (88.7%). Most participants were not an only-child (78.4%) and had a spouse who was not an only-child (55.8%). The most common original family types were nuclear (43.3%) and stem families (48.7%). In terms of occupation, government/public institution/state-owned enterprise (SOE) employees accounted for 54.0%. Long-term migrant workers comprised only 3.3%. Most participants reported being very healthy or healthy with occasional minor illnesses (96.0% for self, 96.2% for family members). Household size of 4–5 persons was the most common (46.0%).

#### 3.1.1 Baseline characteristics of traditional fertility attitude

Among women who held traditional fertility attitudes, the following characteristics were notably more prevalent: being a non-only-child (55.26%); engaging in long-term migrant work (68.97%); having family members with frequent illnesses (71.64%); had a household size of 4–5 persons (56.41%) or 6 persons and above (61.79%); family economic status has a “moderate impact” on the decision to have a second child (69.02%); the cost of supporting older parents is a “moderate impact” (66.83%); the cost of raising a second child is a “moderate impact” (73.19%); husband’s expectations are of “moderate impact” (72.62%) and “high impact” (72.42%); parents-in-law’s expectations are of “moderate impact” (79.30%) and “high impact” (77.41%); parents’ expectations are of “moderate impact” (78.21%) and “high impact” (78.26%); expectations from the social circle are of “moderate impact” (81.80%) and “high impact” (80.97%); societal expectations are of “moderate impact” (82.48%); perceived reimbursement amount from fertility policies is of “moderate impact” (67.21%); and perceived level of community support is of “moderate impact” (71.67%). The specific results were shown in Table 1.

**Table 1:** Univariate analysis of factors influencing traditional fertility attitudes.

Variables	Group	Holding traditional fertility attitudes, *n* (%)		*χ* ^2^	*p*
		Yes	No		
Residence	Urban	718 (52.76)	643 (47.24)	2.89	0.089
	Rural	242 (57.48)	179 (42.52)		
Age	18–29	118 (49.17)	122 (50.83)	4.03	0.133
	30–39	514 (53.37)	449 (46.63)		
	40–45	328 (56.65)	251 (43.35)		
Educational level	Senior secondary or below	108 (55.96)	85 (44.04)	2.12	0.347
	Associate/Bachelor’s degree	148 (57.36)	110 (42.64)		
	Master s degree or above	704 (52.89)	627 (47.11)		
Marital status	Married, without children	107 (52.97)	95 (47.03)	0.07	0.785
	Married, with children	853 (53.99)	727 (46.01)		
Spouse’s only-child status	Yes	414 (52.54)	374 (47.46)	1.01	0.315
	No	546 (54.93)	448 (45.07)		
Only-child status	Yes	188 (48.83)	197 (51.17)	5.02	0.025
	No	772 (55.26)	625 (44.74)		
Original family type	Nuclear family	403 (52.20)	369 (47.80)	3.44	0.487
	Stem family	478 (55.07)	390 (44.93)		
	Joint family	55 (59.78)	37 (40.22)		
	Single-parent family	11 (45.83)	13 (54.17)		
	Blended family	13 (50.00)	13 (50.00)		
Occupation	Government/public institution/state-owned enterprise (SOE)	522 (54.21)	441 (45.79)	0.59	0.964
	Foreign/private enterprise	67 (51.54)	63 (48.46)		
	Farmer/worker (or laborer)	119 (54.09)	101 (45.91)		
	Self-employed/freelance	108 (51.10)	88 (44.90)		
	Other	144 (52.75)	129 (47.25)		
Long-term migrant work status	Yes	40 (68.97)	18 (31.03)	5.51	0.019
	No	917 (53.34)	802 (46.66)		
Self-health status	Very healthy, no illness	364 (49.59)	370 (50.41)	11.68	0.003
	Healthy, occasional minor illness	562 (57.52)	415 (42.48)		
	Frequent illness	34 (47.89)	37 (52.11)		
Family members’ health status	Very healthy, no illness	319 (47.97)	346 (52.03)	21.70	<0.001
	Healthy, occasional minor illness	593 (56.48)	457 (43.52)		
	Frequent illness	48 (71.64)	19 (28.36)		
Household size	2–3	346 (48.26)	371 (51.74)	17.43	<0.001
	4–5	462 (56.41)	357 (43.59)		
	≥6	152 (61.79)	94 (38.21)		
Total household monthly income	Below 6, 000 RMB	342 (56.81)	260 (43.19)	3.30	0.347
	6, 000–10, 000 RMB	308 (52.03)	284 (47.97)		
	10, 000–15, 000 RMB	161 (53.31)	141 (46.69)		
	Above 15,000 RMB	149 (52.10)	137 (47.90)		
Family economic status	Low impact	73 (28.63)	182 (71.37)	91.04	<0.001
	Moderate impact	176 (69.02)	79 (30.98)		
	High impact	711 (55.90)	561 (44.10)		
The cost of supporting older parents	Low impact	191 (38.82)	301 (61.18)	74.55	<0.001
	Moderate impact	274 (66.83)	136 (33.17)		
	High impact	495 (56.25)	385 (43.75)		
The cost of raising a second child	Low impact	88 (30.14)	204 (69.82)	108.49	<0.001
	Moderate impact	202 (73.19)	74 (26.81)		
	High impact	670 (55.19)	544 (44.81)		
Husband’s fertility expectations	Low impact	211 (28.17)	538 (71.83)	343.46	<0.001
	Moderate impact	321 (72.62)	121 (27.38)		
	High impact	428 (72.42)	163 (27.58)		
Parents-in-law’s fertility expectations	Low impact	271 (30.01)	632 (69.99)	419.74	<0.001
	Moderate impact	360 (79.30)	94 (20.70)		
	High impact	329 (77.41)	96 (22.59)		
Parents’ fertility expectations	Low impact	270 (30.00)	630 (70.00)	417.00	<0.001
	Moderate impact	366 (78.21)	102 (21.79)		
	High impact	324 (78.26)	90 (21.74)		
Fertility expectations from the social circle (colleagues, relatives, neighbors, friends)	Low impact	415 (37.29)	698 (62.71)	328.22	<0.001
	Moderate impact	328 (81.80)	73 (18.20)		
	High impact	217 (80.97)	51 (19.03)		
Societal fertility expectations	Low impact	369 (35.65)	666 (64.35)	333.99	<0.001
	Moderate impact	339 (82.48)	72 (17.52)		
	High impact	252 (75.00)	84 (25.00)		
Perceived reimbursement amount from fertility policies	Low impact	257 (39.78)	389 (60.22)	90.81	<0.001
	Moderate impact	330 (67.21)	161 (32.79)		
	High impact	373 (57.83)	272 (42.17)		
Perceived level of community support	Low impact	243 (37.04)	413 (62.96)	139.30	<0.001
	Moderate impact	339 (71.67)	134 (28.33)		
	High impact	378 (57.89)	275 (42.11)		

#### 3.1.2 Baseline characteristics of emerging fertility attitude

Women who held emerging fertility attitudes tend to report: family members’ health status as “very healthy” (54.44%); family economic status as “low impact” (86.67%); the cost of supporting older parents as “low impact” (76.22%); the cost of raising a second child as “low impact” (89.04%); husband’s expectations as “low impact” (73.16%); parents-in-law’s expectations as “low impact” (71.21%); parents’ expectations as “low impact” (71.44%); expectations from the social circle as “low impact” (65.77%); societal expectations as “low impact” (67.83%); perceived reimbursement amount from fertility policies as “low impact” (70.12%); and perceived level of community support as “low impact” (71.49%). The specific results were shown in Table 2.

**Table 2:** Univariate analysis of factors influencing emerging fertility attitudes.

Variables	Group	Holding emerging fertility attitudes, *n* (%)		*χ* ^2^	*p*
		Yes	No		
Residence	Urban	703 (51.65)	658 (48.35)	1.13	0.288
	Rural	205 (48.69)	216 (51.31)		
Age	18–29	127 (52.92)	113 (47.08)	1.09	0.579
	30–39	480 (49.84)	483 (50.16)		
	40–45	301 (51.99)	278 (48.01)		
Educational attainment	Senior secondary or below	105 (54.40)	88 (45.60)	1.24	0.537
	Associate/Bachelor’s degree	127 (49.22)	131 (50.78)		
	Master’s degree or above	676 (50.79)	655 (49.21)		
Marital status	Married, without children	91 (45.05)	111 (54.95)	3.18	0.075
	Married, with children	817 (51.71)	763 (48.29)		
Spouse’s only-child status	Yes	397 (50.38)	391 (49.62)	0.19	0.666
	No	511 (51.41)	483 (48.59)		
Only-child status	Yes	197 (51.17)	188 (48.83)	0.01	0.924
	No	711 (50.89)	686 (49.11)		
Original family type	Nuclear family	398 (51.55)	374 (48.45)	6.92	0.140
	Stem family	447 (51.50)	421 (48.50)		
	Joint family	35 (38.04)	57 (61.96)		
	Single-parent family	13 (54.17)	11 (45.83)		
	Blended family	15 (57.69)	11 (42.31)		
Occupation	Government/public institution/state-owned enterprise (SOE)	487 (50.57)	476 (49.43)	0.15	0.997
	Foreign/private enterprise	67 (51.54)	63 (48.46)		
	Farmer/worker (or laborer)	114 (51.82)	106 (48.18)		
	Self-employed/freelance	100 (51.02)	96 (48.98)		
	Other	140 (51.28)	133 (48.72)		
Long-term migrant work status	Yes	25 (43.10)	33 (56.90)	1.45	0.229
	No	879 (51.13)	840 (48.87)		
Self-health status	Very healthy, no illness	385 (52.45)	349 (47.55)	3.62	0.163
	Healthy, occasional minor illness	494 (50.56)	483 (49.44)		
	Frequent illness	29 (40.85)	42 (59.15)		
Family members’ health status	Very healthy, no illness	362 (54.44)	303 (45.56)	8.96	0.011
	Healthy, occasional minor illness	521 (49.62)	529 (50.38)		
	Frequent illness	25 (37.31)	42 (62.69)		
Household size	2–3	368 (51.32)	349 (48.68)	0.07	0.967
	4–5	415 (50.67)	404 (49.33)		
	≥6	125 (50.81)	121 (49.19)		
Total household monthly income	Below 6, 000 RMB	289 (48.01)	313 (51.99)	6.44	0.092
	6, 000–10, 000 RMB	299 (50.51)	293 (49.49)		
	10, 000–15, 000 RMB	157 (51.99)	145 (48.01)		
	Above 15,000 RMB	163 (56.99)	123 (43.01)		
Family economic status	Low impact	221 (86.67)	34 (13.33)	151.90	<0.001
	Moderate impact	116 (45.49)	139 (54.51)		
	High impact	571 (44.89)	701 (55.11)		
The cost of supporting older parents	Low impact	375 (76.22)	117 (23.78)	174.61	<0.001
	Moderate impact	161 (39.27)	249 (60.73)		
	High impact	372 (42.27)	508 (57.73)		
The cost of raising a second child	Low impact	260 (89.04)	32 (10.96)	202.88	<0.001
	Moderate impact	117 (42.39)	159 (57.61)		
	High impact	531 (43.74)	683 (56.26)		
Husband’s fertility expectations	Low impact	548 (73.16)	201 (26.84)	255.29	<0.001
	Moderate impact	158 (35.75)	284 (64.25)		
	High impact	202 (34.18)	389 (65.82)		
Parents-in-law’s fertility expectations	Low impact	643 (71.21)	260 (28.79)	301.99	<0.001
	Moderate impact	146 (32.16)	308 (67.84)		
	High impact	119 (28.00)	306 (72.00)		
Parents’ fertility expectations	Low impact	643 (71.44)	257 (28.56)	308.29	<0.001
	Moderate impact	153 (32.69)	315 (67.31)		
	High impact	112 (27.05)	302 (72.59)		
Fertility expectations from the social circle (colleagues, relatives, neighbors, friends)	Low impact	732 (65.77)	381 (34.23)	262.15	<0.001
	Moderate impact	114 (28.43)	287 (71.57)		
	High impact	62 (23.13)	206 (76.87)		
Societal fertility expectations	Low impact	702 (67.83)	333 (32.17)	281.71	<0.001
	Moderate impact	118 (28.71)	293 (71.29)		
	High impact	88 (26.19)	248 (73.81)		
Perceived reimbursement amount from fertility policies	Low impact	453 (70.12)	193 (29.88)	149.85	<0.001
	Moderate impact	189 (38.49)	302 (61.51)		
	High impact	266 (41.24)	379 (58.76)		
Perceived level of community support	Low impact	469 (71.49)	187 (28.51)	177.89	<0.001
	Moderate impact	171 (36.15)	302 (63.85)		
	High impact	268 (41.04)	385 (58.96)		

### 3.2 Univariate analysis of factors influencing the fertility attitudes among married reproductive-aged women

The results of univariate analysis showed that whether it was the traditional fertility attitudes was significantly correlated with the following factors: only-child status (*χ*^2^ = 5.02, *p* = 0.025), long-term migrant work status (*χ*^2^ = 5.51, *p* = 0.019), self-health status (*χ*^2^ = 11.68, *p* = 0.003), family members’ health status (*χ*^2^ = 21.70, *p* < 0.001), household size (*χ*^2^ = 17.43, *p* < 0.001), family economic status (*χ*^2^ = 91.04, *p* < 0.001), the cost of supporting older parents (*χ*^2^ = 74.55, *p* < 0.001), the cost of raising a second child (*χ*^2^ = 108.49, *p* < 0.001), husband’s fertility expectations (*χ*^2^ = 343.46, *p* < 0.001), parents-in-law’s fertility expectations (*χ*^2^ = 419.74, *p* < 0.001), fertility expectations from the social circle (colleagues, relatives, neighbors, friends) (*χ*^2^ = 328.22, *p* < 0.001), societal fertility expectations (*χ*^2^ = 333.99, *p* < 0.001), perceived reimbursement amount from fertility policies (*χ*^2^ = 90.81, *p* < 0.001), and perceived level of community support (*χ*^2^ = 139.30, *p* < 0.001). The specific results were shown in Table 1.

Univariate analysis indicated that holding emerging fertility attitudes was significantly associated with family members’ health status (*χ*^2^ = 8.96, *p* = 0.011), the perceived impact of family economic status (*χ*^2^ = 151.90, *p* < 0.001), as well as expectations from spouses, family, social circles, and society, and perceived policy support (all *p* < 0.001). The specific results were shown in Table 2.

### 3.3 Binary logistic regression analysis of factors influencing of fertility attitudes among married reproductive-aged women

#### 3.3.1 Multicollinearity test of independent variables

Before conducting binary logistic regression analysis, multicollinearity diagnostics were performed on the independent variables. The results indicated that the variance inflation factor (VIF) values for all independent variables ranged from 1.04 to 5.70. All VIF values were far below the threshold of 10, indicating no severe multicollinearity issues among the independent variables. This ensures the robustness and reliability of the subsequent regression analysis results.

#### 3.3.2 Binary logistic regression analysis

To clarify the specific effects of various factors on married women’s formation of traditional or emerging fertility attitudes, two binary logistic regression models were constructed. The dependent variables were “whether respondents held traditional fertility attitudes” and “whether respondents held emerging fertility attitudes”. All independent variables were included in the respective models. The variable definitions and coding schemes are presented in Supplementary Tables 2, 3.

As shown in Table 3, educational attainment, family members’ health status, perceived family economic status, husband’s fertility expectations, parents-in-law’s expectations, parents’ expectations, societal fertility expectations, and perceived level of community support were significant factors influencing the holding of traditional fertility attitudes (*p* < 0.05). Specifically, relative to participants with a senior secondary education or below, those with a master’s degree or above exhibited a 0.567-fold decrease in the likelihood of holding traditional fertility attitudes (OR = 0.567, 95% CI: 0.342 ~ 0.940, *p* = 0.028). Compared to participants who perceived their family members’ health as “very healthy,” those with “frequently ill” family members had 2.880 times higher likelihood of holding traditional fertility attitudes (OR = 2.880, 95% CI: 1.83 ~ 5.993, *p* = 0.005). In comparison to those reporting “low impact” of family economic status on fertility decisions, the “moderate impact” group had 1.911 times higher odds of holding traditional fertility attitudes (OR = 1.911, 95% CI: 1.111 ~ 3.289, *p* = 0.019). Groups who perceived their husbands’ fertility expectations as having a “moderate impact” and “high impact” were 1.505 times (OR = 1.505, 95% CI: 1.028 ~ 2.203, *p* = 0.035) and 1.824 times (OR = 1.824, 95% CI: 1.257 ~ 2.647, *p* = 0.002) more likely to hold traditional attitudes, respectively, compared to those in the “low impact” group. Similar patterns were observed for fertility expectations from parents-in-law (“moderate impact”: OR = 1.911, 95% CI: 1.119 ~ 3.266, *p* = 0.018; “high impact”: OR = 2.007, 95% CI: 1.120 ~ 3.596, *p* = 0.019) and parents (“moderate impact”: OR = 1.773, 95% CI: 1.051 ~ 2.991, *p* = 0.032; “high impact”: OR = 1.919, 95% CI: 1.064 ~ 3.461, *p* = 0.030). Compared to those who perceived societal fertility expectations as having a “low impact,” the “moderate impact” group was 1.931 times more likely to hold traditional fertility attitudes (OR = 1.931, 95% CI: 1.223 ~ 3.049, *p* = 0.005). Participants who perceived community support as having a “moderate impact” had 2.203 times higher likelihood of holding traditional fertility attitudes than those perceiving it as having a “low impact” (OR = 2.203, 95% CI: 1.524 ~ 3.184, *p* < 0.001).

**Table 3:** Binary logistic regression analysis of factors influencing traditional fertility attitudes.

Variable	Group	*β*	*p*	OR	95% CI of OR
Residence (Reference: urban)	Rural	0.038	0.844	1.039	0.710 ~ 1.520
Age (Reference: 18-29)	30–39	0.082	0.673	1.085	0.742 ~ 1.586
	40–45	0.164	0.443	1.178	0.776 ~ 1.788
Educational attainment (Reference: senior secondary or below)	Associate/Bachelor’s degree	−0.248	0.313	0.781	0.482 ~ 1.263
	Master’s degree or above	−0.567	0.028	0.567	0.342 ~ 0.940
Marital status (Reference: married, without children)	Married, with children	−0.027	0.894	0.973	0.655 ~ 1.447
Spouse’s only-child status (Reference: yes)	No	0.040	0.742	1.041	0.819 ~ 1.324
Only-child status (Reference: yes)	No	0.254	0.093	1.289	0.959 ~ 1.734
Original family type (Reference: nuclear family)	Stem family	0.085	0.498	1.089	0.851 ~ 1.393
	Joint family	0.007	0.981	1.007	0.583 ~ 1.738
	Single-parent family	−0.433	0.406	0.649	0.234 ~ 1.799
	Blended family	−0.415	0.383	0.661	0.260 ~ 1.676
Occupation (Reference: government/public institution/state-owned enterprise)	Foreign/Private Enterprise	0.096	0.688	1.101	0.688 ~ 1.762
	Farmer/worker (or laborer)	−0.463	0.066	0.630	0.384 ~ 1.031
	Self-employed/freelance	−0.101	0.660	0.904	0.576 ~ 1.418
	Other	−0.283	0.131	0.754	0.523 ~ 1.087
Long-term migrant work status (Reference: yes)	No	−0.313	0.373	0.731	0.367 ~ 1.457
Self-health status (Reference: very healthy, no illness)	Healthy, occasional minor illness	0.098	0.581	1.103	0.778 ~ 1.564
	Frequent illness	−0.558	0.106	0.573	0.291 ~ 1.126
Family members’ health status (Reference: very healthy, no illness)	Healthy, occasional minor illness	0.220	0.222	1.247	0.875 ~ 1.776
	Frequent illness	1.058	0.005	2.880	1.383 ~ 5.993
Household size (Reference: 2-3)	4–5	0.247	0.057	1.280	0.992 ~ 1.651
	≥6	0.318	0.107	1.374	0.934 ~ 2.021
Total household monthly income (Reference: below 6, 000 RMB)	6, 000–10, 000 RMB	−0.072	0.631	0.930	0.692 ~ 1.250
	10, 000–15, 000 RMB	0.091	0.636	1.095	0.751 ~ 1.597
	Above 15,000 RMB	0.047	0.814	1.048	0.709 ~ 1.548
Family economic status (Reference: low impact)	Moderate impact	0.648	0.019	1.911	1.111 ~ 3.289
	High impact	0.368	0.176	1.444	0.848 ~ 2.461
The cost of supporting older parents (Reference: low impact)	Moderate impact	0.115	0.557	1.122	0.763 ~ 1.650
	High impact	0.154	0.411	1.167	0.808 ~ 1.686
The cost of raising a second child (Reference: low impact)	Moderate impact	0.335	0.225	1.398	0.813 ~ 2.403
	High impact	−0.287	0.293	0.751	0.440 ~ 1.281
Husband’s fertility expectations (Reference: low impact)	Moderate impact	0.409	0.035	1.505	1.028 ~ 2.203
	High impact	0.601	0.002	1.824	1.257 ~ 2.647
Parents-in-law’s fertility expectations (Reference: low impact)	Moderate impact	0.648	0.018	1.911	1.119 ~ 3.266
	High impact	0.697	0.019	2.007	1.120 ~ 3.596
Parents’ fertility expectations (Reference: low impact)	Moderate impact	0.573	0.032	1.773	1.051 ~ 2.991
	High impact	0.652	0.030	1.919	1.064 ~ 3.461
Fertility expectations from the social circle (colleagues, relatives, neighbors, friends) (Reference: low impact)	Moderate impact	0.135	0.571	1.145	0.718 ~ 1.826
	High impact	0.510	0.091	1.665	0.922 ~ 3.007
Societal fertility expectations (Reference: low impact)	Moderate impact	0.658	0.005	1.931	1.223 ~ 3.049
	High impact	0.064	0.801	1.066	0.648 ~ 1.754
Perceived reimbursement amount from fertility policies (Reference: low impact)	Moderate impact	0.237	0.200	1.267	0.882 ~ 1.819
	High impact	0.029	0.882	1.030	0.700 ~ 1.516
Perceived level of community support (Reference: low impact)	Moderate impact	0.790	<0.001	2.203	1.524 ~ 3.184
	High impact	0.365	0.062	1.440	0.981 ~ 2.114

As shown in Table 4, economic pressure, social expectations, and policy perception were significant factors influencing the holding of emerging fertility attitudes (*p* < 0.05). Firstly, economic pressure showed a significant suppressive effect, which was particularly evident in the perceived cost of supporting older parents and the cost of raising a second child. Relative to the group perceiving a “low impact” of older adult care expenses on fertility decisions, those perceiving a “moderate impact” and “high impact” were 0.641 times (OR = 0.641, 95% CI: 0.445 ~ 0.925, *p* = 0.017) and 0.616 times (OR = 0.616, 95% CI: 0.434 ~ 0.874, *p* = 0.007) as likely to hold emerging fertility attitudes, respectively. Similarly, compared to the “low impact” group for the cost of raising a second child, the “moderate impact” and “high impact” groups exhibited a 0.389-fold (OR = 0.389, 95% CI: 0.215 ~ 0.703, *p* = 0.002) and 0.356-fold (OR = 0.356, 95% CI: 0.197 ~ 0.643, *p* = 0.001) decrease in the likelihood of holding emerging fertility attitudes, respectively. Secondly, relative to those who perceived parents’ fertility expectations as having a “low impact,” the “high impact” group also showed an inhibitory effect on holding emerging fertility attitudes (OR = 0.504, 95% CI: 0.283 ~ 0.897, *p* = 0.020). Regarding societal fertility expectations, compared to the “low impact” group, the “moderate impact” was associated with 0.564-fold (OR = 0.564, 95% CI: 0.366 ~ 0.869, *p* = 0.009) lower likelihood of holding emerging fertility attitudes. Finally, perceiving government reimbursement amounts from fertility policies as having a “moderate impact” showed a significant inhibitory effect (OR = 0.644, 95% CI: 0.457 ~ 0.909, *p* = 0.012). Perceiving community support as having a “moderate impact” (OR = 0.561, 95% CI: 0.394 ~ 0.796, *p* = 0.001) and “high impact” (OR = 0.669, 95% CI: 0.463 ~ 0.967, *p* = 0.032) also demonstrated significant negative associations.

**Table 4:** Binary logistic regression analysis of factors influencing emerging attitudes.

Variable	Group	*β*	*p*	OR	95% CI of OR
Residence (Reference: urban)	Rural	−0.261	0.168	0.770	0.531 ~ 1.116
Age (Reference: 18-29)	30–39	−0.265	0.157	0.768	0.532 ~ 1.108
	40–45	−0.258	0.215	0.772	0.514 ~ 1.162
Educational attainment (Reference: senior secondary or below)	Associate/Bachelor’s degree	0.087	0.725	1.091	0.672 ~ 1.772
	Master’s degree or above	0.180	0.486	1.197	0.722 ~ 1.984
Marital status (Reference: married, without children)	Married, with children	0.318	0.102	1.374	0.939 ~ 2.011
Spouse’s only-child status (Reference: yes)	No	0.053	0.658	1.054	0.835 ~ 1.331
Only-child status (Reference: yes)	No	−0.046	0.752	0.955	0.719 ~ 1.270
Original family type (Reference: nuclear family)	Stem family	0.035	0.775	1.035	0.816 ~ 1.314
	Joint family	−0.420	0.131	0.657	0.382 ~ 1.133
	Single-parent family	0.417	0.408	1.517	0.565 ~ 4.073
	Blended family	0.445	0.351	1.560	0.613 ~ 3.973
Occupation (Reference: government/public institution/state-owned enterprise)	Foreign/Private Enterprise	−0.064	0.783	0.938	0.596 ~ 1.477
	Farmer/Worker (or Laborer)	0.274	0.268	1.316	0.810 ~ 2.137
	Self-employed/Freelance	0.024	0.917	1.024	0.658 ~ 1.593
	Other	0.145	0.415	1.156	0.815 ~ 1.640
Long-term migrant work status (Reference: yes)	No	−0.168	0.603	0.845	0.448 ~ 1.595
Self-health status (Reference: very healthy, no illness)	Healthy, occasional minor illness	0.281	0.107	1.325	0.941 ~ 1.864
	Frequent illness	−0.068	0.840	0.934	0.483 ~ 1.807
Family members’ health status (Reference: very healthy, no illness)	Healthy, occasional minor illness	−0.233	0.187	0.792	0.560 ~ 1.120
	Frequent illness	−0.491	0.162	0.612	0.308 ~ 1.219
Household size (Reference: 2-3)	4–5	0.032	0.798	1.033	0.807 ~ 1.322
	≥6	0.294	0.127	1.341	0.920 ~ 1.955
Total household monthly income (Reference: below 6, 000 RMB)	6, 000–10, 000 RMB	−0.030	0.837	0.970	0.727 ~ 1.294
	10, 000–15, 000 RMB	−0.068	0.713	0.934	0.649 ~ 1.345
	Above 15,000 RMB	0.161	0.404	1.175	0.805 ~ 1.715
Family economic status (Reference: low impact)	Moderate impact	−0.463	0.136	0.630	0.343 ~ 1.157
	High impact	−0.315	0.298	0.730	0.403 ~ 1.321
The cost of supporting older parents (Reference: low impact)	Moderate impact	−0.445	0.017	0.641	0.445 ~ 0.925
	High impact	−0.485	0.007	0.616	0.434 ~ 0.874
The cost of raising a second child (Reference: low impact)	Moderate impact	−0.945	0.002	0.389	0.215 ~ 0.703
	High impact	−1.033	0.001	0.356	0.197 ~ 0.643
Husband’s fertility expectations (Reference: low impact)	Moderate impact	−0.237	0.215	0.789	0.543 ~ 1.148
	High impact	−0.245	0.195	0.783	0.540 ~ 1.134
Parents-in-law’s fertility expectations (Reference: low impact)	Moderate impact	−0.204	0.449	0.815	0.480 ~ 1.383
	High impact	−0.347	0.239	0.707	0.397 ~ 1.260
Parents’ fertility expectations (Reference: low impact)	Moderate impact	−0.486	0.065	0.615	0.367 ~ 1.031
	High impact	−0.685	0.020	0.504	0.283 ~ 0.897
Fertility expectations from the social circle (colleagues, relatives, neighbors, friends) (Reference: low impact)	Moderate impact	−0.221	0.326	0.802	0.516 ~ 1.247
	High impact	−0.171	0.550	0.843	0.481 ~ 1.476
Societal fertility expectations (Reference: Low impact)	Moderate impact	−0.573	0.009	0.564	0.366 ~ 0.869
	High impact	−0.404	0.095	0.668	0.415 ~ 1.074
Perceived reimbursement amount from fertility policies (Reference: Low impact)	Moderate impact	−0.440	0.012	0.644	0.457 ~ 0.909
	High impact	−0.266	0.157	0.766	0.529 ~ 1.108
Perceived level of community support (Reference: low impact)	Moderate impact	−0.579	0.001	0.561	0.394 ~ 0.796
	High impact	−0.402	0.032	0.669	0.463 ~ 0.967

## 4 Discussion

This study presents the differentiation of fertility attitudes among married reproductive-aged women and provides an analysis of the factors associated with holding of traditional versus emerging fertility attitudes. The findings indicated that the formation of traditional fertility attitudes was primarily related to health status, fertility expectations from family members, societal fertility expectations and community support. Concurrently, emerging fertility attitudes are significantly associated with internal family economic pressure, certain external social expectations, and perceived inadequacy of policy and community support. This indicates that in the contemporary fertility decisions of reproductive-aged women, traditional and emerging fertility attitudes do not represent a simple mutual substitution. Instead, they form a complex, interwoven network of associations that jointly characterize reproductive intentions and behaviors.

### 4.1 Factors associated with traditional fertility attitudes

#### 4.1.1 Health risk

The results of this study showed that the health status of family members is an important factor associated with the formation of traditional fertility attitudes. Compared to women who perceived their family members as “very healthy,” those who perceived their family members as “frequently ill” had a significantly higher likelihood of holding traditional fertility attitudes. To some extent, this reflects the traditional fertility ideas of “raising sons for old age security” and “more sons, more happiness” - the notion that having more children is perceived to increase a family’s ability to cope with future risk, thereby providing greater future security. This finding is in line with theoretical perspectives on risk response and family security (30, 31). Some prior studies have suggested that the act of giving birth not only has the function of emotional comfort, but is also regarded by some families as a long-term investment with the attribute of “raising sons for old age security” (30). That is, when a family faces health risks, its members may be more inclined to hold traditional fertility attitudes to build a more robust family support network to cope with the uncertainty regarding future care resources. This mechanism aligns with the social security perspective, which posits that formal security systems can partially substitute for family care functions (31), thereby weakening traditional fertility attitudes. Thus, in contexts where families feel vulnerable due to health concerns and perceive formal supports as insufficient, traditional fertility attitudes may be prominent as a compensatory risk-coping strategy.

#### 4.1.2 Family expectations

Existing research has indicated that in Chinese families, the fertility motivation of reproductive-aged women is not purely an individual decision but is also significantly associated with the expectations of family members (32–34). This study found that family expectations were also associated with the orientation of fertility attitudes to a certain extent. Family expectations operate through distinct dynamics. On the one hand, in China, the fertility expectations of husbands are often related to traditional family role attitudes (35). Some studies have pointed out a phenomenon of “reproductive dependence” among married women in China, meaning that the fertility intentions of some women are more closely aligned with their husbands’ concepts and expectations, even exceeding their original reproductive tendencies (36, 37). This presents a normative pressure on women and is thus associated with their acceptance of traditional fertility attitudes. On the other hand, the fertility expectations of parents-in-law and parents carry the ethical responsibilities associated with “filial piety” culture and “continuing the family line” (38, 39). In Chinese family ethics, the concept that “among the three forms of unfilial conduct, having no descendants is the gravest” has a profound presence. Consequently, when women face these intense expectations rooted in intergenerational ethics, they often must navigate a balance between “respecting elders’ wishes” and “maintaining family harmony.” This dynamic is related to their tendency to accept and internalize the corresponding traditional fertility attitudes. In conclusion, the intra-familial expectations constitute a critical layer of correlated factors that must be accounted for in understanding the diversity of fertility attitudes.

#### 4.1.3 Societal expectations

The results of this study indicated that societal fertility expectations have a significant positive association with traditional fertility attitudes among married reproductive-aged women. This finding suggests that social public opinion and cultural norms are aligned with women’s fertility cognitions to a certain extent. In traditional Chinese fertility culture, notions such as “more sons, more happiness” and “continuing the family line” are not only values held within families but also important standards for society to evaluate a woman’s role and family worth (27). Consequently, the perceived pressure of social evaluation presents as a prominent factor. Research has indicated that some contemporary women still tend to realize their personal social value through childbearing, especially when they perceive strong societal expectations. To avoid being labeled as “unfilial” or “irresponsible,” they may be more inclined to accept and internalize traditional fertility attitudes (36, 40). This relationship suggests that fertility attitudes are not merely individual choices but are also closely linked to the social and cultural environment. This view is also consistent with the findings from both domestic and international studies (26, 41). Consequently, it is implied that efforts aiming to promote the modernization of fertility attitudes should consider the effect of the socio-cultural environment.

#### 4.1.4 Community support

The statistical results indicated that the perceived support from the community in terms of systems, policies and welfare for having children by the participants had a significant positive association with traditional fertility attitudes. This finding highlights the evolving role of communities as critical grassroots units connecting macro policies and micro families, particularly within the contemporary context of transitioning toward a fertility-friendly society (42). Take Guangdong, which has long been the province with the highest fertility rate in China, as an example (43). This coexists not only from its strong traditional views on fertility, but also from the specific measures taken at the community level in recent years to reduce the burden of childbirth, such as providing childbirth subsidies and offering universal childcare services (43). This suggests that, as society transitions toward being more fertility-friendly, effective implementation of community support policies at the grassroots level may help sustain traditional fertility beliefs such as “more sons, more happiness.”

### 4.2 Factors associated with emerging fertility attitudes

#### 4.2.1 Older adult care costs

The results of this study indicate that the cost of supporting older parents is significantly and negatively associated with emerging fertility attitudes among married reproductive-aged women. This phenomenon can be explained through the theory of intergenerational resource competition. Under the constraints of finite household economic resources, expenditures for older adult care coexist with investments in child-rearing. Existing research has pointed out that in the context of accelerating family aging, the medical expenses of the older adult are often accompanied by a reduction in resources allocated to children’s education (44). This trade-off is linked to more cautious cognitive assessments under the dual pressure of raising children and supporting the older adult, thereby pointing to lower acceptance of emerging fertility attitudes that emphasize intensive child-rearing and elite education. This relationship highlights that contemporary family fertility attitudes are deeply embedded within the economic reality of intergenerational resource allocation. Consequently, it is implied that the financial burden of older adult care acts as a critical structural constraint, suggesting that mitigating these intergenerational resource conflicts could be a relevant consideration in efforts aimed at promoting the modernization of fertility attitudes.

#### 4.2.2 Childrearing expenditure

The results of this study indicated that the perceived cost of child-rearing is significantly and negatively associated with emerging fertility attitudes among married reproductive-aged women. This finding indicates that the high childrearing costs constitute a critical economic barrier coexisting with a slower transition of fertility attitudes from emphasizing quantity to prioritizing quality. This relationship can be explained by the cost–benefit theory. One of the characteristics of emerging fertility attitudes is the shift from pursuing more children to investing in higher child quality (26), the realization of which is highly dependent on sufficient family economic resources. In modern society, parents’ sense of responsibility and their perceived economic risks are related to risk-averse fertility decisions (45), and the persistent decline in fertility rates coexists partly with the inability to provide superior upbringing conditions (such as housing, education, medical care and so on) for children (46). Besides, mothers facing significant family financial burdens may adjust their parenting styles in relation to childrearing costs (47). These phenomena further illustrate that the high economic costs associated with raising children are tied not only to lower willingness to have children but also to significant challenges to emerging fertility attitudes such as “quality child-rearing” and “elite education.” Consequently, it is implied that the modernization of fertility attitudes remains heavily dependent on economic realities, suggesting that mitigating the immediate financial pressures of childbearing, childrearing, and education could be a relevant consideration for facilitating cognitive shifts.

#### 4.2.3 Parental expectations

This study also found that fertility expectations from parents were significantly and negatively associated with emerging fertility attitudes among married reproductive-aged women. In German families, the social pressure exerted by parents can deeply intersect with personal fertility orientations (48). This negative relationship can be explained through intergenerational ethical pressure. In China, most parents with a child aged over 18 were born before 1985. These parental expectations often carry traditional fertility attitudes such as “continuing the family line” and “more sons, more happiness” (38, 39). When parents hold strong expectations for grandchildren, this may create an implicit ethical pressure on women through emotional bonds and norms of responsibility, potentially prompting them to place greater consideration on complying with elders’ wishes in fertility decisions. Consequently, this is linked to a reduction in the space for emerging fertility attitudes centered on “high-quality childbirth and upbringing” or personal development. This view is consistent with previous studies (49, 50), which indicate that Chinese parental influences are associated with women’s fertility attitudes through cultural norms and societal perceptions. Consequently, it is implied that addressing these subtle intergenerational pressures could be a relevant consideration for facilitating shifts in fertility cognitions.

#### 4.2.4 Societal expectations

The results of this study indicate that perceived societal fertility expectations also have a significant negative association with emerging fertility attitudes among married reproductive-aged women. It suggests that widespread public discourse and cultural norms may carry a diffuse form of pressure, which may simultaneously coexist with lower levels of individual identification with emerging fertility attitudes centered on high-quality childbirth and upbringing. This negative relationship can be interpreted from the perspective of normative compliance. In the socio-cultural context of China, childbearing is not merely a personal choice but also an expression of family responsibility and social obligation (27). When women perceive intense societal expectations for childbearing, they may feel compelled to align their attitudes with traditional fertility norms to avoid being labeled as failing in their family duties or deviating from social expectations. Previous research has indicated that fertility consciousness can be deeply shaped by social structures and environments (45). This conclusion has been further verified in this study. Consequently, it is implied that the modernization of fertility attitudes remains anchored within the pervasive socio-cultural atmosphere, suggesting that fostering a more diverse and accommodating cultural environment regarding reproductive orientations could be a relevant consideration for facilitating cognitive shifts.

### 4.3 Recommendations for future research on fertility attitudes

It is worth noting that contemporary demographic research faces a major conceptual and methodological challenge: the lack of clear theoretical definitions and standardized measurement tools to distinguish between traditional and emerging fertility attitudes. This study is no exception, particularly regarding the single-item measure of emerging attitudes and the unclear dimensional relationship between the two constructs. Therefore, this study strongly recommends that future research in psychometrics and sociology prioritize the development of validated, multi-item scales specifically designed to measure fertility-related attitudes. Future studies need to clarify whether emerging fertility attitudes represent an entirely opposing extreme dimension to traditional values, are independent dimensions, or partially overlapping frameworks. Establishing a clear and empirically validated questionnaire system would not only help resolve current conceptual ambiguities but also significantly enhance the reliability of research on fertility transitions.

## 5 Study strengths and limitations

This study had several strengths. Firstly, it adopted a multi-stage sampling method, ensuring the randomness and representativeness across urban, rural, and different administrative regions. Secondly, this study collected 1,782 valid questionnaires. The sufficient sample size ensured the reliability of subsequent statistical analyses. Thirdly, the research perspective was innovative. In contrast to the majority of existing studies that focus on fertility intentions and single influencing factors, this study took fertility attitudes as the core research object, explored the differences and factors in the association of the two types of attitudes, and enriched the theoretical research on fertility sociology. Fourthly, the measurement tools demonstrated good reliability and validity. The traditional fertility attitude scale had excellent internal consistency (Cronbach’s *α* = 0.941), and the measurement of emerging fertility attitudes also showed criterion-related validity consistent with the theory.

Despite the above strengths, this study has several inherent limitations. Firstly, the cross-sectional design only investigated fertility attitudes and influencing factors at a specific point in time, which cannot deeply reflect their dynamic changes alongside social policies, economic environments, and individual circumstances. Therefore, future research should adopt a longitudinal design to capture these dynamic changes. Secondly, the research scope was limited to Shandong Province, a coastal region in eastern China. Given the significant socioeconomic and cultural differences between China’s coastal and inland regions, the findings may not be directly generalizable to inland provinces. Therefore, future research should conduct multi-regional surveys to enhance cross-regional generalizability and extend the investigation to inland regions to capture the full spectrum of fertility attitude differentiation across China. Thirdly, although the multi-stage sampling method was employed, this study did not formally compare the sample distribution with census data or other official statistics of Shandong Province due to the lack of directly comparable variables/classifications, and thus the representativeness of the sample for all married women of childbearing age in Shandong remains unverified. Consequently, future research should optimize the sampling strategy to achieve a more representative occupational distribution and incorporate population-based weighting procedures and representativeness assessments. Fourthly, the definition of fertility attitudes was based on relevant literature and operationalized using questionnaire items. Although the traditional fertility attitude scale demonstrated acceptable reliability and validity, the emerging fertility attitude was measured using a single indicator, which may not fully capture the multidimensional nature of fertility attitudes. Therefore, future research should develop and validate a multi-item scale for fertility attitudes to reflect the multidimensional nature of fertility attitudes. Fifthly, statistical analysis of this study treated the dataset under the assumption of simple random sampling, neglecting the inherent clustering effects and sampling weights. Such an approach may lead to underestimated standard errors and exaggerated significance levels. Therefore, accounting for complex survey designs remains a critical requirement for future studies.

## 6 Conclusion

This study indicates that the fertility attitudes of married reproductive-aged women do not constitute a simple dichotomy between traditional and modern perspectives. Instead, they present a complex interplay where the two coexist and intertwine. The findings indicate that traditional fertility attitudes show a significant positive association with factors such as family health risks, fertility expectations from family members and society, and perceived community support. This reflects the potential relevance of family security needs, intergenerational ethics, and social norms in the continuation of traditional fertility culture. In addition, emerging fertility attitudes show a significant negative association with the financial burden of older adult care and childrearing expenditures, certain societal expectations, and perceived inadequacy of existing policy support. This highlights that high costs of eldercare and parenting, coupled with perceived structural pressures, coexist with a slower transition toward modern fertility attitudes focused on “child quality enhancement.” These collective findings indicate that the fertility attitudes of contemporary women are a dynamic evaluation process embedded within the intersecting contexts of tradition and modernity, family and society, economy and culture.

## Data Availability

The raw data supporting the conclusions of this article will be made available by the authors, without undue reservation.

## References

[1] Nations U World Population Prospects 2022 (2022) Available online at: https://www.un-ilibrary.org/content/books/9789210014380 (Accessed October 29, 2025).

[2] Chen C Xiong X Tang G. A decomposition study on the factors influencing China's total fertility rate changes between 1990 and 2020. Sci Rep. (2024) 14:28176. doi: 10.1038/s41598-024-79999-4, 39548321 PMC11567968

[3] Wang F Zhao L Zhao Z. China’s family planning policies and their labor market consequences. J Popul Econ. (2017) 30:31–68. doi: 10.1007/s00148-016-0613-0

[4] Run G Xinzheng S. How does the universal two-child policy affect fertility behavior? China Econ Q Int. (2023) 3:227–37. doi: 10.1016/j.ceqi.2023.11.002

[5] Shi K Cui W Chen S Zhang X Wang X Cao M . Effect of “universal two-child” policy on population changes in Shandong province, China: an interrupted time series analysis. Front Public Health. (2025) 13:1612141–1. doi: 10.3389/fpubh.2025.1612141, 40951382 PMC12423096

[6] Huang X. Can China's two-child policy reverse fertility decline? An in-depth analysis of fertility behavior and desires. Afr J Reprod Health. (2025) 29:74–90. doi: 10.29063/ajrh2025/v29i9.8, 41048190

[7] Xiang Z Zhang X Li Y Li J Wang Y Ming WK . Fertility intention and its affecting factors in China: a national cross-sectional survey. Heliyon. (2023) 9:e13445. doi: 10.1016/j.heliyon.2023.e13445, 36814608 PMC9939585

[8] Ren Y Cheng X Niu C Yang F Zheng Y. Fertility intention of young people of childbearing age in China after the implementation of the two-child policy-a systematic review and meta-analysis. BMC Public Health. (2024) 24:3518. doi: 10.1186/s12889-024-20956-1, 39695553 PMC11657275

[9] Liu X Cheng L Zhang T Bi L Lu J Wen M . Fertility intention and its influencing factors among young people of reproductive age in China: a cross-sectional survey study. Acta Psychol. (2026) 262:106169. doi: 10.1016/j.actpsy.2025.106169, 41601125

[10] Zhou Y Bian Y. Why has China’s fertility rate plummeted in the past decade? An investigation of fertility intentions and influencing factors among single, unmarried women of childbearing age in China. Soc Sci. (2025) 14:293. doi: 10.3390/socsci14050293

[11] yu PW. Research on the construction of new fertility concepts in the context of population change. J Shandong Women's Univ. (2025) 2:140–54.

[12] Yufei W Kuili S. From post-70s to post-00s: an intergenerational comparative study on fertility motivations of Chinese women. Jingchu Academic Journal. (2025) 26:91–101. doi: 10.14151/j.cnki.jcxk.2025.05.013

[13] Xia Z. Current situation and countermeasures of youth's fertility view under the background of the three-child fertility policy. Beijing Youth Res. (2022) 31:28–33. doi: 10.3969/j.issn.1008-4002.2022.02.004

[14] Ji Y. Between tradition and modernity: “leftover” women in Shanghai. J Marriage Fam. (2015) 77:1057–73. doi: 10.1111/jomf.12220

[15] Yu Y Rongxin H Ning Z Liming L. Second-child fertility intentions among urban women in China: a systematic review and Meta-analysis. Int J Environ Res Public Health. (2023) 20:3744–4. doi: 10.3390/ijerph20043744, 36834437 PMC9962327

[16] Raymo JM Park H Xie Y Yeung WJJ. Marriage and family in East Asia: continuity and change. Annu Rev Sociol. (2015) 41:471–92. English. doi: 10.1146/annurev-soc-073014-112428, 30078932 PMC6070151

[17] Qiu T. Does urban housing affordability affect fertility in China. J Popul Res. (2025) 42:47–7. doi: 10.1007/s12546-025-09402-0

[18] Chen J Zhou XC. Sandwich generation in China: exchange pattern with older parents and educational expenditure on young children. Asian J Soc Sci. (2022) 50:122–9. doi: 10.1016/j.ajss.2022.01.007

[19] Dong W-H Wang X Yuan F Wang L Gu T-M Zhu B-Q . Will a government subsidy increase couples’ further fertility intentions? A real-world study from a large-scale online survey in eastern China. Human Reprod Open. (2024) 2024:hoae055. doi: 10.1093/hropen/hoae055, 39421541 PMC11484797

[20] Yi Y Erxun Z Yingying Z. Study on the couplling relationship between population migration and economic development along the eastern section of Mount Huangshan Mountain. Econ Res Guid. (2024) 8:13–8. doi: 10.3969/j.issn.1673-291X.2024.08.004

[21] Zhike J Zhihua L. Regional variations of the fertility-intention-and-behavior deviation among Chinese young women: findings from the cultural and socioeconomic perspectives. Chin J Popul Sci. (2024) 38:66–81.

[22] Zhang C Sobotka T. Drastic changes in fertility level and timing in response to marriage and fertility policies: evidence from Shandong province, China. China Popul Dev Stud. (2021) 5:191–214. doi: 10.1007/s42379-021-00089-3

[23] Yang S Jiang Q Sánchez-Barricarte JJ. China’s fertility change: an analysis with multiple measures. Popul Health Metrics. (2022) 20:12. doi: 10.1186/s12963-022-00290-7, 35361257 PMC8969406

[24] Karimollah H-T. Sample size estimation in epidemiologic studies. Casp J Intern Med. (2011) 2:289–98. PMC3895825 24551434

[25] Cui W Guo X Shi K Cao M Zhou Z Hao H . Second birth intentions and its influencing factors among reproductive-aged women: a cross-sectional study conducted in Shandong Province, China. Front Public Health. (2025) 13:1665360. doi: 10.3389/fpubh.2025.1665360, 41221241 PMC12597926

[26] Youhua C Yongjian S. Fertility culture, fertility system and fertility rate: the effect of the three-child policy and its supporting measures. J Hohai Univ. (2024) 26:12–28. doi: 10.3876/j.issn.1671-4970.2024.01.002

[27] Yongping L. On traditional fertility culture. Chin Cult Res. (1996) 2:23–28+147.

[28] Jing L Hongping L. Research on the evolution of fertility concept in China and its transformation and development in the new era. Popul Soc. (2024) 40:81–93. doi: 10.14132/j.2095-7963.2024.06.007

[29] Xiaojun Z Yuan Z Chunmiao P Hanfeng L Yucheng X Menglan Z . Knowledge of infectious disease prevention and control among school physicians in Futian District, Shenzhen, and influencing factors. Chin J Sch Doctor. (2024) 38:636–720. doi: 10.20161/j.cnki.32-1199/R.202409177

[30] Yuanfa T Li Q. The impact of child quantity and quality on subjective well-being of the elderly. Res Develop Western China. (2020) 1:57–73.

[31] Lifei Z Zichen X Zhi X Wei C. Establishing a sound social security system and the fertility desire of residents: from the perspective of long-term care insurance. J Financ Res. (2025) 11:58–76.

[32] Jian S Bo H. Fertility motivation and intention of Chinese people of childbearing age. Population Econ. (2022) 6:1–16. doi: 10.3969/j.issn.1000-4149.2022.00.052

[33] Tianyuan T Yiwen C Xinyue L. First-marriage couples' fertility motivation and fertility intention: evidence from the City of Yinchuan. Academic J Jinyang. (2025) 5:54–64. doi: 10.16392/j.cnki.14-1057/c.2025.05.007

[34] Jing Z Hui Y. Analysis of factors influencing third-child fertility intention among the childbearing-age population. Statistics Decision. (2022) 38:72–7. doi: 10.13546/j.cnki.tjyjc.2022.20.014

[35] Menghan Z Xiaohan Z Yongai J. How intergenerational coresidence affects first-child fertility intention. Popul Res. (2025) 49:36–50. doi: 10.12451/202603.02940

[36] Sijing C Zhen W Shasha Y Peng Z Quan H. Fertility dependence or fertility autonomy? The impact of husbands' traditional gender role conceptions on wives' fertility intentions. Acta Psychol Sin. (2025) 57:1661–76. doi: 10.3724/SP.J.1041.2025.1661, 42213867

[37] Shisong Q Jiahao W Xi L. Gender bargaining in family fertility decision-making and the realization of fertility intention. Popul Res. (2025) 49:115–28. doi: 10.12451/202603.02945

[38] Chaofan X Chao G. The impact of filial piety concepts on fertility behavior and policy implications. Social Sciences of Beijing. (2024) 10:116–28. doi: 10.13262/j.bjsshkxy.bjshkx.241010

[39] Heyang Z. The Influence of Dual Filial Piety Concepts on Fertility Intentions: An Empirical Study Based on CGSS Data: Guizhou University. (2025) Available online at: https://link.cnki.net/doi/10.27047/d.cnki.ggudu.2025.002173 (Accessed November 11, 2025).

[40] Jianming H Kefei Z. The influence of gender role concept on young people's fertility intention. Popul J. (2025) 47:43–59. doi: 10.16405/j.cnki.1004-129X.2025.05.003

[41] Mera S. Assessment of socio cultural factors affecting the utilization of family planning services: the case of Haramaya town bate kebele. Res Humanit Soc Sci. (2019) 9:48–53. doi: 10.7176/RHSS/9-21-06

[42] Zhao Z Wei X. Improving the fertility support policy system to promote high-quality population development. China Population News. (2025). doi: 10.28125/n.cnki.ncrcb.2025.000102

[43] Sixuan L Hanneng L Jinhui H. Stirring the 'pool of spring water' in population work: Guangdong tops nation in births and resident population growth for 2024; only province with over 1 million births for five consecutive years. Nanfang: Southern daily (2025).

[44] Limin S Shiqi Y. Family aging, intergenerational resource transfer and investment in children's education. Popul Dev. (2025) 31:151–60.

[45] Juan F Xiaoyu L. Strategies for addressing China's fertility challenges from the perspective of the Marxist view of life. Future Develop. (2025) 50:1–12.

[46] Jiayan G Chuanhua Y. China's population changes and thoughts on health countermeasures. J Public Health Prev Med. (2026) 37:1–7. doi: 10.3969/j.issn.1006-2483.2026.01.001

[47] Lianping W Wanhong D Huiqin Z Danhua X Lifang C. Study on partum and its influencing factors in difference stages of puerperium. Shanghai Nurs. (2022) 22:37–42. doi: 10.3969/j.issn.1009-8399.2022.05.008

[48] Kuhnt A-K Trappe H. Channels of social influence on the realization of short-term fertility intentions in Germany. Adv Life Course Res. (2016) 27:16–29. doi: 10.1016/j.alcr.2015.10.002

[49] Lihong Z Yingnan W. The impact of the "universal two-child" policy on population development in Liaoning Province. Co-Operative Econ Sci. (2016) 16:155–8. doi: 10.13665/j.cnki.hzjjykj.2016.16.073

[50] Yan L Ling Z Bo H. Intergenerational transmission of fertility motivation-based on CFPS 2020 data analysis. Popul Dev. (2025) 31:79–89.

