# Supplementary material for: Differentiation of fertility attitudes and its influencing factors among married reproductive-aged women: an empirical analysis in Shandong Province, China
Source: Front Public Health. 2026 Jun 10;14:1840600. doi: 10.3389/fpubh.2026.1840600 (PMC13290774; doi:10.3389/fpubh.2026.1840600)
Supplement: Supplementary file 1 [file Table_1.docx]

**Supplementary Table:**

| **Supplementary Table 1. Baseline characteristics of the total sample (n=1782)** | | |
| --- | --- | --- |
| Variable | Group | n (%) |
| Residence | Urban | 1361 (76.4) |
|  | Rural | 421 (23.6) |
| Age | 18-29 | 240 (13.5) |
|  | 30-39 | 963 (54.0) |
|  | 40-45 | 579 (32.5) |
| Educational level | Senior secondary or below | 193 (10.8) |
|  | Associate/Bachelor’s degree | 258 (14.5) |
|  | Master’s degree or above | 1331 (74.7) |
| Marital status | Married, without children | 202 (11.3) |
|  | Married, with children | 1580 (88.7) |
| Spouse’s only-child status | Yes | 788 (44.2) |
|  | No | 994 (55.8) |
| Only-child status | Yes | 385 (21.6) |
|  | No | 1397 (78.4) |
| Original family type | Nuclear family | 772 (43.3) |
|  | Stem family | 868 (48.7) |
|  | Joint family | 92 (5.2) |
|  | Single-parent family | 24 (1.3) |
|  | Blended family | 26 (1.5) |
| Occupation | Government / Public Institution / State-Owned Enterprise (SOE) | 963 (54.0) |
|  | Foreign / Private Enterprise | 130 (7.3) |
|  | Farmer / Worker (or Laborer) | 220 (12.3) |
|  | Self-employed / Freelance | 196 (11.0) |
|  | Other | 273 (15.3) |
| Long-term migrant work status | Yes | 58 (3.3) |
|  | No | 1724 (96.7) |
| Self-health status | Very healthy, no illness | 734 (41.2) |
|  | Healthy, occasional minor illness | 977 (54.8) |
|  | Frequent illness | 71 (4.0) |
| Family members’ health status | Very healthy, no illness | 665 (37.3) |
|  | Healthy, occasional minor illness | 1050 (58.9) |
|  | Frequent illness | 67 (3.8) |
| Household size | 2-3 | 717 (40.2) |
|  | 4-5 | 819 (46.0) |
|  | ≥6 | 246 (13.8) |
| Total household monthly income | Below 6, 000 RMB | 602 (33.8) |
|  | 6, 000-10, 000 RMB | 592 (33.2) |
|  | 10, 000-15, 000 RMB | 302 (16.9) |
|  | Above 15,000 RMB | 286 (16.0) |
| **Continued Supplementary Table 1** | | |
| Variable | Group | n (%) |
| Family economic status | Low impact | 255 (14.3) |
|  | Moderate impact | 255 (14.3) |
|  | High impact | 1272 (71.4) |
| The cost of supporting older parents | Low impact | 492 (27.6) |
|  | Moderate impact | 410 (23.0) |
|  | High impact | 880 (49.4) |
| The cost of raising a second child | Low impact | 292 (16.4) |
|  | Moderate impact | 276 (15.5) |
|  | High impact | 1214 (68.1) |
| Husband’s fertility expectations | Low impact | 749 (42.0) |
|  | Moderate impact | 442 (24.8) |
|  | High impact | 591 (33.2) |
| Parents-in-law’s fertility expectations | Low impact | 903 (50.7) |
|  | Moderate impact | 454 (25.5) |
|  | High impact | 425 (23.8) |
| Parents’ fertility expectations | Low impact | 900 (50.5) |
|  | Moderate impact | 468 (26.3) |
|  | High impact | 414 (23.2) |
| Fertility expectations from the social circle (colleagues, relatives, neighbors, friends) | Low impact | 1113 (62.5) |
|  | Moderate impact | 401 (22.5) |
|  | High impact | 268 (15.0) |
| Societal fertility expectations | Low impact | 1035 (58.1) |
|  | Moderate impact | 411 (23.1) |
|  | High impact | 336 (18.8) |
| Perceived reimbursement amount from fertility policies | Low impact | 646 (36.3) |
|  | Moderate impact | 491 (27.6) |
|  | High impact | 645 (36.2) |
| Perceived level of community support | Low impact | 656 (36.8) |
|  | Moderate impact | 473 (26.5) |
|  | High impact | 653 (36.6) |

| **Supplementary Table 2. Variable definitions for the binary logistic regression analysis on the influencing factors of traditional fertility attitudes** | |
| --- | --- |
| Variables | Definitions |
| Residence | 1=Urban; 2=Rural |
| Age | 1=18-29; 2=30-39; 3=40-45 |
| Educational attainment | 1=Senior secondary or below; 2= Associate/Bachelor’s degree; 3= Master’s degree or above |
| Marital status | 1=Married, without children; 2= Married, with children |
| Spouse’s only-child status | 1=Yes; 2=No |
| Only-child status | 1=Yes; 2=No |
| Original family type | 1=Nuclear family; 2=Stem family; 3=Joint family; 4=Single-parent family; 5= Blended family |
| Occupation | 1=Government / Public Institution / State-Owned Enterprise (SOE); 2=Foreign / Private Enterprise; 3=Farmer / Worker (or Laborer); 4=Self-employed / Freelance; 5=Other |
| Long-term migrant work status | 1=Yes; 2=No |
| Self-health status | 1=Very healthy, no illness; 2=Healthy, occasional minor illness; 3=Frequent illness |
| Family members’ health status | 1=Very healthy, no illness; 2=Healthy, occasional minor illness; 3=Frequent illness |
| Household size | 1=2-3; 2=4-5; 3=≥6 |
| Total household monthly income | 1=Below 6, 000 RMB; 2=6, 000-10, 000 RMB; 3=10, 000-15, 000 RMB; 4= Above 15,000 RMB |
| Family economic status | 1=Low impact; 2=Moderate impact; 3=High impact |
| The cost of supporting older parents | 1=Low impact; 2=Moderate impact; 3=High impact |
| The cost of raising a second child | 1=Low impact; 2=Moderate impact; 3=High impact |
| Husband’s fertility expectations | 1=Low impact; 2=Moderate impact; 3=High impact |
| Parents-in-law’s fertility expectations | 1=Low impact; 2=Moderate impact; 3=High impact |
| Parents’ fertility expectations | 1=Low impact; 2=Moderate impact; 3=High impact |
| Fertility expectations from the social circle (colleagues, relatives, neighbors, friends) | 1=Low impact; 2=Moderate impact; 3=High impact |
| Societal fertility expectations | 1=Low impact; 2=Moderate impact; 3=High impact |
| Perceived reimbursement amount from fertility policies | 1=Low impact; 2=Moderate impact; 3=High impact |
| Perceived level of community support | 1=Low impact; 2=Moderate impact; 3=High impact |

| **Supplementary Table 3. Variable definitions for the binary logistic regression analysis on the influencing factors of emerging fertility attitudes** | |
| --- | --- |
| Variables | Definitions |
| Residence | 1=Urban; 2=Rural |
| Age | 1=18-29; 2=30-39; 3=40-45 |
| Educational attainment | 1=Senior secondary or below; 2= Associate/Bachelor’s degree; 3= Master’s degree or above |
| Marital status | 1=Married, without children; 2= Married, with children |
| Spouse’s only-child status | 1=Yes; 2=No |
| Only-child status | 1=Yes; 2=No |
| Original family type | 1=Nuclear family; 2=Stem family; 3=Joint family; 4=Single-parent family; 5= Blended family |
| Occupation | 1=Government / Public Institution / State-Owned Enterprise (SOE); 2=Foreign / Private Enterprise; 3=Farmer / Worker (or Laborer); 4=Self-employed / Freelance; 5=Other |
| Long-term migrant work status | 1=Yes; 2=No |
| Self-health status | 1=Very healthy, no illness; 2=Healthy, occasional minor illness; 3=Frequent illness |
| Family members’ health status | 1=Very healthy, no illness; 2=Healthy, occasional minor illness; 3=Frequent illness |
| Household size | 1=2-3; 2=4-5; 3=≥6 |
| Total household monthly income | 1=Below 6, 000 RMB; 2=6, 000-10, 000 RMB; 3=10, 000-15, 000 RMB; 4= Above 15,000 RMB |
| Family economic status | 1=Low impact; 2=Moderate impact; 3=High impact |
| The cost of supporting older parents | 1=Low impact; 2=Moderate impact; 3=High impact |
| The cost of raising a second child | 1=Low impact; 2=Moderate impact; 3=High impact |
| Husband’s fertility expectations | 1=Low impact; 2=Moderate impact; 3=High impact |
| Parents-in-law’s fertility expectations | 1=Low impact; 2=Moderate impact; 3=High impact |
| Parents’ fertility expectations | 1=Low impact; 2=Moderate impact; 3=High impact |
| Fertility expectations from the social circle (colleagues, relatives, neighbors, friends) | 1=Low impact; 2=Moderate impact; 3=High impact |
| Societal fertility expectations | 1=Low impact; 2=Moderate impact; 3=High impact |
| Perceived reimbursement amount from fertility policies | 1=Low impact; 2=Moderate impact; 3=High impact |
| Perceived level of community support | 1=Low impact; 2=Moderate impact; 3=High impact |
